# Delayed Presentation of a Nonunion of the Distal Femur Associated With a Missed Popliteal Artery Injury: A Case Report

**DOI:** 10.7759/cureus.40642

**Published:** 2023-06-19

**Authors:** Xavier Penda, Henry Ndasi, Ghislain Aminake, Serge Tima

**Affiliations:** 1 Orthopaedics and Traumatology, Baptist Hospital Mutengene, Mutengene, CMR

**Keywords:** multidisciplinary approach, cardiac arrest, distal femur nonunion, delayed presentation, popliteal injury

## Abstract

Popliteal artery injuries are known complications of distal femur fractures, often leading to life-threatening consequences. The authors present a case of a four-month-old missed popliteal artery injury complicating a nonunion of the distal femur. The patient, a 28-year-old male, initially sought alternative treatment after a bike accident but experienced persistent pain and functional limitations. During our clinical evaluation, the possibility of a popliteal artery injury was considered but not definitively ruled out. However, during surgery, an incomplete transection of the popliteal artery was discovered, posing life-threatening complications. Fortunately, through effective management, we were able to address the complications and proceed with the planned procedure. This case underscores the importance of recognizing vascular damage in delayed fracture presentations and highlights the necessity of a prompt, multidisciplinary approach to handle unexpected surgical complications. Insights gained from this case contribute to raising awareness and preparedness among healthcare providers in similar challenging scenarios.

## Introduction

Popliteal artery injuries following distal femur or proximal tibia fractures [1,2] are well-documented acute complications that can result in life-threatening bleeding and limb-threatening consequences if left untreated [3]. Prompt recognition and early intervention are crucial to prevent devastating complications. However, delayed presentations of popliteal artery injury following femur fractures are rarely reported in the literature. We present a challenging case of a 28-year-old male patient who sought consultation after a four-month-old trauma involving a bike accident. Despite the initial loss of consciousness, the patient opted for alternative treatment from a bone settler. With persistent pain and unsuccessful outcomes, he eventually presented to our institution. This case showcases the complexity of managing a nonunion of the distal femur and an unexpected popliteal artery injury. Herein, we present our successful management approach, highlighting the surgical procedure and postoperative care.

## Case presentation

**Figure 1:**
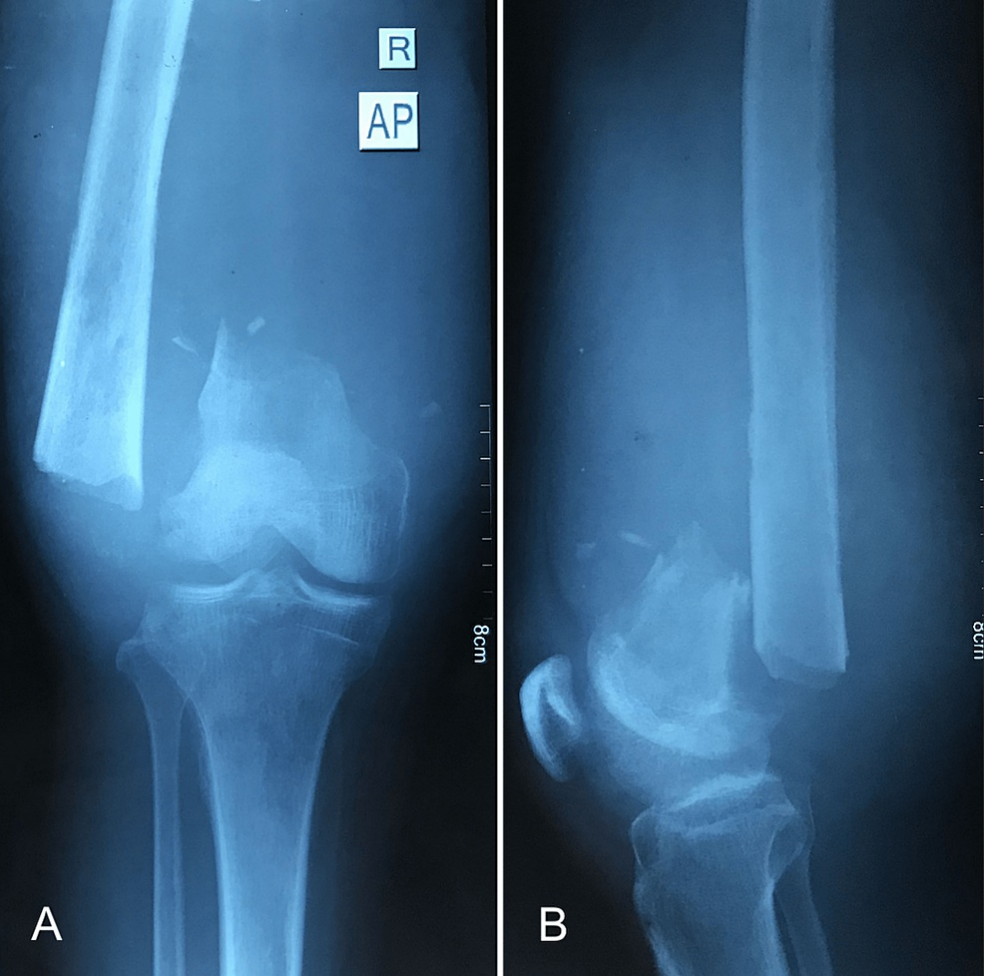
Preoperative plain radiographs of the right knee Distal femur nonunion surrounded with fluid (A) Anteroposterior view; (B) Lateral view

Upon presentation, the patient was non-weight-bearing on the right limb, accompanied by a markedly enlarged distal thigh, measuring nearly twice the size of the contralateral side. The palpation revealed a substantial collection in the distal thigh, without signs of compartment syndrome or local inflammation. The distal pulses were palpable. Neurologically, the patient demonstrated decreased sensation distal to the knee and a foot drop. A puncture of the collection revealed reddish, old blood. A radiographic evaluation (Figure 1) confirmed the presence of a distal femur nonunion, and aside from anemia (hemoglobin level of 8.6 g/dl), the laboratory tests were unremarkable, reducing the likelihood of infection.

After evaluation, we determined that the patient was presenting with a nonunion of a distal femur fracture, accompanied by foot drop and reduced sensation below the knee. Additionally, there was a surrounding hematoma from the initial injury.

The patient could not afford any electromyographic exploration at the time and preferred to address the nonunion prior to other investigations.

Plan and surgical procedure

After a thorough explanation of the surgical procedure, associated risks, and informed consent, the patient was scheduled for an open reduction and internal fixation of the distal femur. We opted for a retrograde intramedullary nail with a side plate due to the short distal fragment. The surgical approach involved a lateral approach to the distal femur, performed under spinal anesthesia with the patient in a supine position. During the procedure, a significant amount of aged blood clots and uncoagulated blood (approximately 1 L) were encountered. Progressive and meticulous debridement was performed until active heavy bleeding was identified, originating from the popliteal artery. Due to the challenging exposure and bleeding control, a decision was made to clamp the femoral artery proximally via an inguinal transverse approach.

Intraoperative complication and management:

Unfortunately, during the approach of the femoral artery, the patient experienced a cardiac arrest. Prompt cardio-pulmonary resuscitation was initiated, and the anesthetic team successfully stabilized the patient. Subsequently, the exploration continued, identifying a 1 cm long incomplete transection of the popliteal artery, which was meticulously repaired using a running suture with Prolene 4/0. Following the release of the femoral clamp, the suture repair was confirmed to be intact and impermeable.

Fracture fixation and artery repair

**Figure 2:**
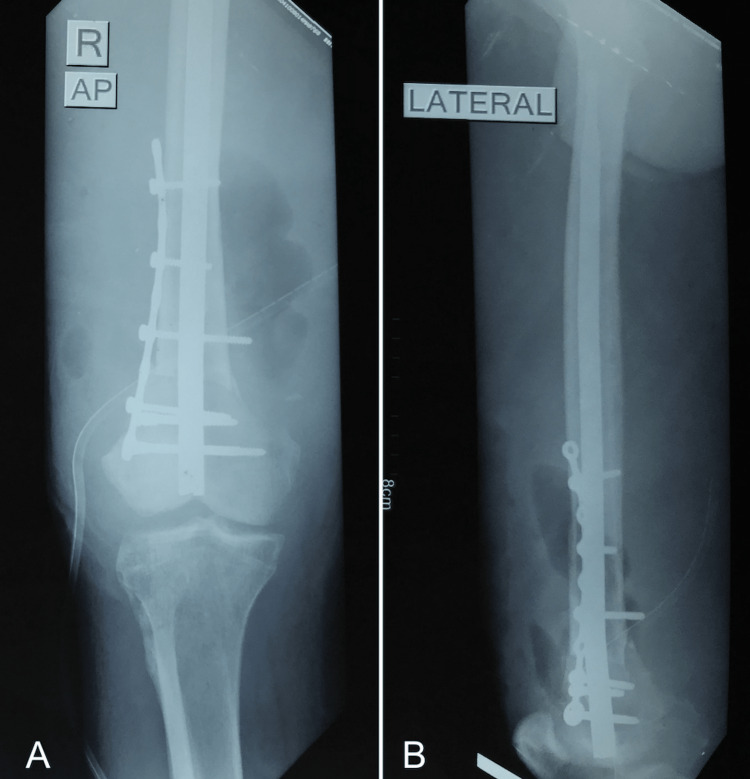
Postoperative plain radiographs of the right knee Internal fixation with a retrograde IM Nail and side plate. (A) Anteroposterior. (B) Lateral.

With the arterial injury successfully managed, we proceeded with the planned femur fixation. To facilitate the fracture reduction and ensure tension-free artery repair, a 1.5 cm segment of the proximal femur was resected (Figure 2).

Thorough wound irrigation was performed, and the incision was closed in layers with the placement of a drain.

Postoperative course and rehabilitation

Postoperatively, the patient was closely monitored in the intensive care unit for two days. Anticoagulant therapy was initiated and continued for one month to mitigate the risk of thromboembolic complications. The patient started ambulation on postoperative Day 5, and a comprehensive rehabilitation program was initiated to optimize functional recovery. In the long term, he developed a deep surgical site infection at two months post-op treated with suppressive antibiotherapy initially. Fortunately, the fracture healed at four months, and we removed the hardware at one year.

## Discussion

A popliteal artery transection injury is a condition that can have severe consequences, including limb necrosis or amputation [4], and it requires urgent management to prevent life-threatening complications [3]. In the present case, we encountered a unique scenario where a delayed presentation of popliteal artery injury occurred following a distal femur fracture that had not healed. This rare combination of a nonunion fracture, a significant hematoma, and an incomplete popliteal artery transection posed a complex challenge in terms of diagnosis and management.

Our case involved a 28-year-old male patient who initially sought alternative treatment from a bone settler after a bike accident. This decision led to persistent pain and an inability to bear weight, prompting him to eventually present to our institution four months after the initial trauma. The delayed presentation highlights a common issue in developing countries [5,6], where appropriate initial care may be lacking, and patients may resort to unqualified practitioners or traditional healers [7,8]. Difficulties related to medical care access in developing countries exacerbate these challenges. Patients may encounter barriers such as limited resources, inadequate healthcare infrastructure, and a lack of awareness about proper medical care. These issues underscore the importance of addressing healthcare disparities, improving access to quality medical care, and educating both healthcare providers and the general public in developing countries [8].

Surgeons should be aware of the possibility of incomplete major artery transections, even in cases with delayed presentations. In this particular case, the patient exhibited an atypical presentation characterized by a massive hematoma, foot drop, and decreased sensation distal to the knee. These findings should raise a high level of suspicion for associated vascular injury. The rarity of delayed presentations of incomplete transections of major arteries makes it crucial for surgeons to maintain vigilance and consider the possibility of vascular damage.

A quick, fast, and multidisciplinary approach is essential when unexpected complications arise during surgical procedures [9]. In our case, the patient experienced a cardiac arrest during the approach of the femoral artery, necessitating prompt cardiopulmonary resuscitation.

Our case report highlights the rarity and complexity of managing a nonunion of a distal femur fracture accompanied by a missed popliteal artery injury. The case emphasizes the importance of considering vascular damage in cases of long bone fractures, even in delayed presentations. Surgeons should maintain a high level of suspicion and be prepared to manage unexpected complications during surgeries. A quick, fast, and multidisciplinary approach, coupled with effective communication and prompt response, is crucial in ensuring successful outcomes in challenging cases. The lessons learned from this case can contribute to the awareness and preparedness of healthcare providers in similar scenarios.

## Conclusions

This case report presents a unique and challenging scenario of a delayed popliteal artery injury accompanying a nonunion of the distal femur. The rarity of this presentation emphasizes the need for increased awareness among healthcare providers and the public regarding the potential risks of seeking alternative treatments without proper medical evaluation and care. Surgeons should remain vigilant and consider the possibility of vascular damage, especially in delayed presentations with unusual features.

## References

[1] Gupta M, Vora HJ, Patil SP, Pundkare GT (2016). Delayed presentation of popliteal artery transection following undisplaced lateral condyle fracture of tibia. J Orthop Allied Sci.

[2] Liu YW, Li YH, Yu T, Yang T, Li Y, Tan L (2020). Popliteal artery transection associated with a minimally displaced tibial plateau fracture: a case report and review of the literature. BMC Musculoskelet Disord.

[3] Reid JJ, Kremen TJ Jr, Oppenheim WL (2013). Death after closed adolescent knee injury and popliteal artery occlusion: a case report and clinical review. Sports Health.

[4] Diouf AB, Dembélé B, Sarr L, Daffé M, Penda X, Diémé C (2017). Fracture of the tibial plateau with lesions of the poplite artery and the sciatic-nerve external poplite about a case and review of the literature. SM J Orthop.

[5] Agarwal-Harding KJ, Chokotho LC, Mkandawire NC, Martin C Jr, Losina E, Katz JN (2019). Risk factors for delayed presentation among patients with musculoskeletal injuries in Malawi. J Bone Joint Surg Am.

[6] Melkamu S, Belay W, Maru L (2022). Proportion and associated factors of delayed presentation among patients with open fracture at Tibebe Ghion Specialized Hospital, Bahir Dar, north west Ethiopia. J Orthop Sports Med.

[7] Card EB, Obayemi JE, Shirima O (2020). Practices and perspectives of traditional bone setters in northern Tanzania. Ann Glob Health.

[8] Yempabe T, Edusei A, Donkor P, Buunaaim A, Mock C (2023). Traditional bonesetters in northern Ghana: opportunities for engagement with the formal health sector. Pan Afr Med J.

[9] Cooper N, Roshdy M, Sciarretta JD, Kaufmann C, Duncan S, Davis J, Macedo FI (2018). Multidisciplinary team approach in the management of popliteal artery injury. J Multidiscip Healthc.

